# Impaired rRNA synthesis triggers homeostatic responses in hippocampal neurons

**DOI:** 10.3389/fncel.2013.00207

**Published:** 2013-11-11

**Authors:** Anna Kiryk, Katharina Sowodniok, Grzegorz Kreiner, Jan Rodriguez-Parkitna, Aynur Sönmez, Tomasz Górkiewicz, Holger Bierhoff, Marcin Wawrzyniak, Artur K. Janusz, Birgit Liss, Witold Konopka, Günther Schütz, Leszek Kaczmarek, Rosanna Parlato

**Affiliations:** ^1^Nencki Institute of Experimental Biology, Polish Academy of SciencesWarsaw, Poland; ^2^DKFZ-ZMBH Alliance, Department of Molecular Biology of the Cell I, German Cancer Research CenterHeidelberg, Germany; ^3^Department of Brain Biochemistry, Institute of Pharmacology, Polish Academy of SciencesCracow, Poland; ^4^Department of Molecular Neuropharmacology, Institute of Pharmacology, Polish Academy of SciencesCracow, Poland; ^5^Institute of Anatomy and Cell Biology, University of HeidelbergHeidelberg, Germany; ^6^Department of Biophysics, Warsaw University of Life Sciences-SGGWWarsaw, Poland; ^7^DKFZ-ZMBH Alliance, Department of Molecular Biology of the Cell II, German Cancer Research CenterHeidelberg, Germany; ^8^Institute of Applied Physiology, University of UlmUlm, Germany

**Keywords:** rRNA, nucleolus, learning and memory, hippocampus, mTOR, neurogenesis

## Abstract

Decreased rRNA synthesis and nucleolar disruption, known as nucleolar stress, are primary signs of cellular stress associated with aging and neurodegenerative disorders. Silencing of rDNA occurs during early stages of Alzheimer's disease (AD) and may play a role in dementia. Moreover, aberrant regulation of the protein synthesis machinery is present in the brain of suicide victims and implicates the epigenetic modulation of rRNA. Recently, we developed unique mouse models characterized by nucleolar stress in neurons. We inhibited RNA polymerase I by genetic ablation of the basal transcription factor TIF-IA in adult hippocampal neurons. Nucleolar stress resulted in progressive neurodegeneration, although with a differential vulnerability within the CA1, CA3, and dentate gyrus (DG). Here, we investigate the consequences of nucleolar stress on learning and memory. The mutant mice show normal performance in the Morris water maze and in other behavioral tests, suggesting the activation of adaptive mechanisms. In fact, we observe a significantly enhanced learning and re-learning corresponding to the initial inhibition of rRNA transcription. This phenomenon is accompanied by aberrant synaptic plasticity. By the analysis of nucleolar function and integrity, we find that the synthesis of rRNA is later restored. Gene expression profiling shows that 36 transcripts are differentially expressed in comparison to the control group in absence of neurodegeneration. Additionally, we observe a significant enrichment of the putative serum response factor (SRF) binding sites in the promoters of the genes with changed expression, indicating potential adaptive mechanisms mediated by the mitogen-activated protein kinase pathway. In the DG a neurogenetic response might compensate the initial molecular deficits. These results underscore the role of nucleolar stress in neuronal homeostasis and open a new ground for therapeutic strategies aiming at preserving neuronal function.

## Introduction

Protein synthesis is essential in the consolidation of long-term memory by long-lasting changes of synapses (Cajigas et al., 2010). Neuronal stimulation and prolonged neuronal activity increase protein synthesis by controlling nucleolar number and/or by regulating processing and maturation of rRNA (Jordan et al., 2007).

In line with a crucial role in learning and memory, rRNA transcription is down-regulated during ageing in CA1 and dentate gyrus (DG) hippocampal areas although in CA3 it is increased to compensate the functional loss in DG and CA1. Interestingly, CA1 and DG are especially vulnerable to brain injury and Alzheimer's disease (AD), supporting the connection between neuronal activity, metabolism, and functional deficits in aged hippocampal neurons (Garcia Moreno et al., 1997). For example, rRNA transcription is decreased in hippocampal neurons after alcohol consumption and deprivation in rats and it is associated with memory impairment (Garcia-Moreno et al., 2001). Recent studies have shown that rRNA transcription is specifically down-regulated in the hippocampus of AD and suicide subjects by epigenetic modifications (McGowan et al., 2008; Pietrzak et al., 2011). Hence, a better understanding of the cellular and molecular strategies adopted to counteract nucleolar stress could pave the way to neuroprotective strategies preserving neuronal function.

The mammalian target of rapamycin (mTOR) is an evolutionarily conserved serine/threonine kinase and an important regulator of cell growth and protein translation, dependent on cellular metabolic state and stress conditions (Laplante and Sabatini, 2012). mTOR dysregulation is associated with several neurodevelopmental and neurodegenerative disorders making its targets and regulators valuable for therapeutic interventions (Crino, 2011). In agreement with its fundamental role, several feedback mechanisms intervene to control its function. For example in response to permissive growth conditions mTOR regulates protein synthesis by controlling rRNA transcription (Mayer et al., 2004; Grewal et al., 2007). In turn, inhibition of rRNA transcription and disruption of nucleolar integrity down-regulate mTOR activity (Rieker et al., 2011).

To elucidate the role of rRNA transcription and nucleolar activity in learning and memory, we have used a genetic approach to induce nucleolar stress in specific neurons. This method is based on the conditional ablation of the nucleolar transcription factor TIF-IA, crucial for recruiting RNA polymerase I (Pol I) to the rDNA promoter (Parlato et al., 2008). Genetic loss of TIF-IA impairs rRNA transcription, causing disruption of nucleoli resulting in severe growth deficits during embryonic development (Yuan et al., 2005). Several intracellular pathways converge on TIF-IA in response to either favorable or adverse conditions. As such TIF-IA activity is differentially regulated by various kinases including mTOR, ERK, AMPK, JNK (Zhao et al., 2003; Mayer et al., 2004, 2005; Hoppe et al., 2009) in response to environmental changes. Recently it has been shown that brain-derived neurotrophic factor (BDNF) regulates Pol I activity by ERK1/2 and TIF-IA activation stimulating hippocampal neuron morphogenesis (neurite length and branching) (Gomes et al., 2011).

Here, we have analyzed behavioral, cellular, and molecular changes in TIF-IA^CaMKCreERT2^ mutants characterized by the inducible ablation of TIF-IA gene in adult hippocampal neurons. Nucleolar stress, as a consequence of the inhibition of rRNA synthesis, results in hippocampal neurodegeneration (Parlato et al., 2008). However, the mutant mice do not show deficits in learning and memory behavioral tests. On the contrary, mutant mice present enhanced spatial learning, suggesting compensatory mechanisms. Moreover, hippocampal neurodegeneration is restricted and nucleolar integrity is restored. Increased neurogenesis in the DG could partially account for the lack of behavioral and morphological deficits. The molecular analysis suggests the activation of neuroprotective strategies to inhibit apoptosis and enhance survival. The mTOR pathway appears to be upregulated in the TIF-IA^CaMKCreERT2^ mutants, supporting the role of nucleolar activity in neuronal homeostasis.

## Materials and methods

### Mice

Homozygous TIF-IA^flox/flox^; CaMKCreERT2 (TIF-IA^CaMKCreERT2^) mutant mice were generated by crossing mice carrying the *TIF-IA* floxed allele (TIF-IA^flox/flox^) to the transgenic line CaMKCreERT2. TIF-IA^+/flox^; CaMKCreERT2 positive mice were again crossed with TIF-IA^flox/flox^ mice. The analysis of the genotype was performed as previously described (Parlato et al., 2008). 2-month-old TIF-IA^flox/flox^ control and TIF-IA^CaMKCreERT2^ mutant mice were both injected intraperitoneally with 1 mg tamoxifen twice a day for five consecutive days and were analyzed at different time points after the last injection, in general by a 2–3 month interval. Both male and female mice were used for the experiments. The mouse genetic background was C57Bl6/N. The experiments were performed and analyzed blind with regard to the mouse genotype.

Procedures involving animal care were approved by the Committee on Animal Care and Use (Regierungspräsidium Karlsruhe) in accordance with the local Animal Welfare Act and the European Communities Council Directive of 24 November 1986 (86/609/EEC).

### Behavioral analysis

The mice were tested at three time points: 5 weeks (controls, *n* = 16: 8 females and 8 males; mutants, *n* = 15: 5 females and 10 males), 3 months (controls, *n* = 6: 2 females and 4 males; mutants, *n* = 5: 2 females and 3 males), and 6 months (controls, *n* = 6: 4 females and 2 males; mutants, *n* = 7: 4 females and 3 males) after tamoxifen. Mice were subjected to behavioral examination in a battery of tests: handling, neurological assessment, Rotarod test, Open Field, Elevated Plus Maze, and Morris water maze test. In the last day of 1-week handling the mice were exposed to neurological assessment in order to find abnormalities in activity, reflexes and sense response, according to a table of neurological deficits (Katz et al., 1998).

For Rotarod test a standard apparatus (TSE systems, Bad Homburg, Germany) was used. Mice were habituated to the immobile rod (day 1) and then to the rod turning with slow speed 4 rpm (day 2). In the 3rd day the test was performed and the rod was turning with increasing speed from 4 to 40 rpm. Time to fall from a rod was measured by the TSE system software.

For the Open Field, mice were placed in the center of gray round arena (60 cm in diameter) surrounded by high walls (30 cm high). The floor of the apparatus was virtually divided to 16 zones in EthoVision system (Noldus Information Technology, Wageningen, The Netherlands). The number of zones crossed by each mouse, the distance moved per minute of test, the total distance and time spent in center and periphery were also measured.

The level of anxiety was assessed in the Elevated Plus Maze test. The plastic apparatus consists of four arms (30 × 5.5 cm each) connected by central area (5.5 × 5.5 cm). The mice were placed individually in the central area and were allowed to move freely for 5 min. Time spent in each zone was measured by EthoVision system.

Morris water maze test was used for assessing hippocampus-dependent memory. In the first day mice were allowed to swim in a pool with visible platform (15 cm high, 11.5 × 11.5 cm) to assess visual and motoric abilities. The procedure was performed as previously described (Widy-Tyszkiewicz et al., 1993; Giese et al., 1998). Each mouse was exposed to 1 training session daily divided for 4 trials in a circular white pool (diameter 1.4 m) with the platform submerged 1.5 cm under water (24–27°C) in position 1. Each mouse was trained to find a hidden platform in a latency of 60 s. If mouse found a platform in 60 s or if the time elapsed, mouse was allowed to stay on the platform for 60 s. Next, memory of platform position 1 was measured in 60 s probe trials 24 and 48 h after the 4th day of training. Mouse was put in the opposite quadrant to “platform quadrant” and was allowed freely swimming for 60 s. After the second probe trail, 1-day training was performed to investigate the ability of re-learning to new platform location—position 2. The swim path and the time spent in four virtual quadrants of the pool were recorded by the EthoVision software.

### Electrophysiology

For *in vitro* long-term potentiation (LTP) recording animals 1 month after tamoxifen were anaesthetized with isoflurane and decapitated. The brains were quickly removed and placed in cold artificial cerebrospinal fluid (aCSF: NaCl 117 mM, MgSO_4_ 1.2 mM, KCl 4.7 mM, CaCl_2_ 2.5 mM, NaHCO_3_ 25 mM, NaH_2_PO_4_ 1.2 mM, 10 mM glucose, bubbled with carbogen). Both hemispheres were cut into 400 μm coronal slices on a vibratome. Slices containing the hippocampus were placed in a recording interface chamber to recover for at least 1.5 h before recording. The slices were continuously perfused with carbogenated aCSF at 33°C. Field excitatory postsynaptic potentials (fEPSPs) were recorded using glass micropipettes filled with 1 M NaCl yielding resistance of 2–3 MΩ, in the stratum radiatum in CA1, evoked by stimulation of the Schaffer collateral-commissural afferents every 30 s with bipolar metal electrodes (test pulses at 0.033 Hz, 0.1 ms). Input-output (I-O) analysis was conducted to determine the maximal response to constant increase. During I-O protocol 16 stimuli with increasing intensity from 0 to maximum value were delivered. Intensity of the test stimulus was adjusted to obtain fEPSP with slopes that were one-third of the maximal response (established on the basis of I-O curve). Paired pulse facilitation experiments were performed before baseline recordings. PPF was examined at inter-stimulus intervals of 15, 25, 35, 50, 100, 150, and 200 ms and stimulus intensity one-third of the maximal response. PPF (%) was determined as a ratio of the second fEPSP slope to the first evoked. After at least 15 min of stable baseline LTP was induced tetanically (three trains of 100 Hz 1 s stimulation, separated by 3 min). After the end of the tetanic protocol, a test pulse was subsequently applied for at least 120 min. Recordings were amplified and digitized and amplitudes of fEPSP were analyzed on-line and off-line. For analysis of LTP, the response slopes were expressed as a percentage of the average response slopes during the baseline period prior to LTP induction.

### Gene expression profiling

Array analysis of gene expression was performed on adult TIF-IA^CaMKCreERT2^ mutant mice and control littermates (*n* = 5–6 per each group) four weeks after injection of tamoxifen. Mice were killed by CO_2_, the brains were immediately removed and preserved in RNA-later solution (Ambion). Total RNA was isolated from hippocampal vibratome sections (300 μm) using the RNeasy Mini Kit (Qiagen). Profiling was performed using the MouseWG-6 v2 BeadChip arrays (Illumina Inc., San Diego, CA, USA) according to the manufacturer's instructions. Microarray quality control was performed using the BeadArray R package from the Bioconductor suite (Gentleman et al., 2004). After background subtraction, the data were normalized using quantile normalization and then log2-transformed. Statistical analysis of differences in transcription was performed with limma from the Bioconductor suite. Ontology analyses were carried out using gene set enrichment analysis (GSEA) and identification of enriched putative transcription factor binding sites was performed using cREMaG. The data were stored in the GEO database (http://www.ncbi.nlm.nih.gov/geo/), record number GSE51240.

### Histological analysis

Mice were sacrificed by CO_2_ and brains were immediately dissected. For immunohistochemistry, brains were fixed in 4% paraformaldehyde overnight and paraffin embedded or sectioned on a vibratome (50 μm thickness), with the exception of paraffin sections that were 7 μm thick. Sections from the region of the hippocampus comprised between Bregma −1.46 mm and −2.70 mm were incubated with primary antibodies overnight at 4°C. Visualization of antigen-bound primary antibodies was carried out using either a biotinylated secondary antibody together with the avidin-biotin system and the VECTOR peroxidase kit (Vector Laboratories). Mice were intraperitoneally injected with 5-bromo-2-deoxyuridine (BrdU; Sigma) 40 mg/kg body weight in 0.1 M Tris and then killed 2 h after a single BrdU injection (*n* = 3–4), or 2 weeks (*n* = 4), and 6 weeks (*n* = 4) after the last of 5 days of one daily BrdU injection. Primary antibodies for immunostaining were: anti-doublecortin (Santa Cruz), anti-bromodeoxyuridine (Dako), anti-Ki67, anti-nucleolin (Abcam), anti-nucleophosmin (NPM/B23), anti-neuronal-specific nuclear protein NeuN (Millipore), anti-phosphoS6 (S235/236) (Cell Signaling), anti-TIF-IA (Abnova). Non-radioactive *in situ* hybridization was performed on paraffin sections as previously described using a specific riboprobe hybridizing to regions in the leader sequence of the pre-rRNA (Parlato et al., 2008).

### RT-qPCR

Total RNA was isolated from dissected hippocampi as for the gene expression profiling. Levels of TIF-IA mRNA and pre-rRNA were monitored by reverse transcription (RT) followed by quantitative PCR (qPCR). Synthesis of cDNA with M-MLV Reverse Transcriptase (Life Technologies) was primed with random hexamers and qPCR was performed on a Light Cycler 480 instrument (Roche) using SYBR Green. TIF-IA mRNA primers (5′-GAACCCACTGAAGATCTGCCTGC and 5′-GGAGTCTCCACCAGCGGTGCTG) were located in exons 14 and 15, respectively. The amplicon is disrupted upon Cre-mediated recombination due to deletion of exon 14. For detection of pre-rRNA, the first 130 nucleotides relative to the transcription start site were amplified using the 5′-ACTGACACGCTGTCCTTTCC and 5′-GACAGCTTCAGGCACCGCGA primers; a primer pair covering the first processing site was also used: 5′-CGTGTAAGACATTCCTATCTCG and 5′- GCCCGCTGGCAGAACGAGAAG. To amplify GAPDH we used the following: 5′-CATGGCCTTCCGTGTTCCTA and 5′-GCGGCACGTCAGATCCA. TaqMan qPCR was performed using the Chromo4 Platform (Biorad). The following TaqMan inventoried gene expression assays were used: 18S rRNA (Mm03928990_g1), Hprt (Mm00446968_m1) (Applied Biosystems/Life Technologies). Hypoxanthine-phophoribosyltransferase (*Hprt*) was chosen as a housekeeping gene. Expression changes were calculated as a fold change vs. mean of control samples.

### Statistical analysis

Behavioral and electrophysiological results were analyzed in Statistica 6.0 (StatSoft). One-Way ANOVA was used for Rotarod, Open Field, Elevated plus maze test and Two-Way ANOVA (genotype and gender) for relearning task in Morris water maze. For multiple comparisons in Morris water maze, the results were at first normalized to equalize variances in statistical program JMP v.5.0.1.2 followed by Two-Way ANOVA with repeated measures. Significant genotype effect was further analyzed using *post-hoc* NIR test. For electrophysiological recordings ANOVA with repeated measures was used. *P* < 0.05 was acknowledged as a significance threshold. Data were pooled across animals of the same genotype and presented as mean ± SEM using GraphPad Prism (GraphPad Software).

The ImageJ program was used to measure DG area and CA1 thickness, namely the average height of the CA1 layer. The DG area per section was measured on average in six coronal paraffin sections (one every 4th sections) per mouse (*n* = 5). CA1 thickness was measured on average in six coronal sections (one every 4th sections) per mouse (*n* = 5). In each section the CA1 thickness was measured in three different regions between the distal and proximal CA1. The number of phosho-S6 positive neurons in DG was measured as the number of double positive phospho-S6 and NeuN cells per section. In CA1 the number of phosho-S6 positive neurons in each section was counted between the distal and proximal CA1 in three area units used as a reference. The number of Ki67, BrdU, doublecortin positive cells in DG was measured as the average number of positive cells per section in five-six coronal vibratome sections (one every 3rd sections) per mouse (*n* = 4–5). For BrdU colocalization, the cells showing a nuclear BrdU staining (green) and surrounded by the doublecortin (brown) staining were counted in the DG. The results of the *t*-test are reported as follows: (^*^p < 0.05; ^**^p < 0.01; ^***^p < 0.001).

## Results

### Impaired rRNA synthesis results in aberrant synaptic plasticity

**Figure 1:**
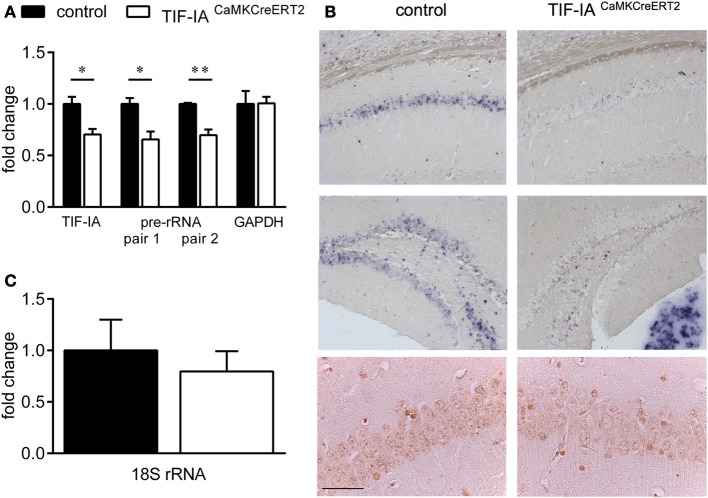
**Conditional ablation of TIF-IA reduces 47S pre-rRNA level in the hippocampus. (A)** TIF-IA and GAPDH mRNA and pre-rRNA decrease by qPCR in the hippocampi of control and TIF-IA^CaMKCreERT2^ mutants 1 month after tamoxifen. **(B)** Upper panels: Representative images showing the specific effects of TIF-IA loss on pre-rRNA synthesis (blue staining) in CA1 and DG by *in situ* hybridization in coronal paraffin sections of control and TIF-IA^CaMKCreERT2^ mutant mice 1 month after tamoxifen. Lower panels: TIF-IA protein analyzed by immunohistochemistry (IHC) in CA1 is visible in the nucleoli of controls but not in TIF-IA^CaMKCreERT2^ mutant mice. **(C)** 18S rRNA analyzed by qPCR in control and TIF-IA^CaMKCreERT2^ mutants 1 month after tamoxifen is not affected; *n* = 5. Error bars represent SEM. Scale bar: **(B)** upper panels: 150 μm; **(B)** lower panels: 60 μm. ^*^p < 0.05; ^**^p < 0.01.

**Figure 2:**
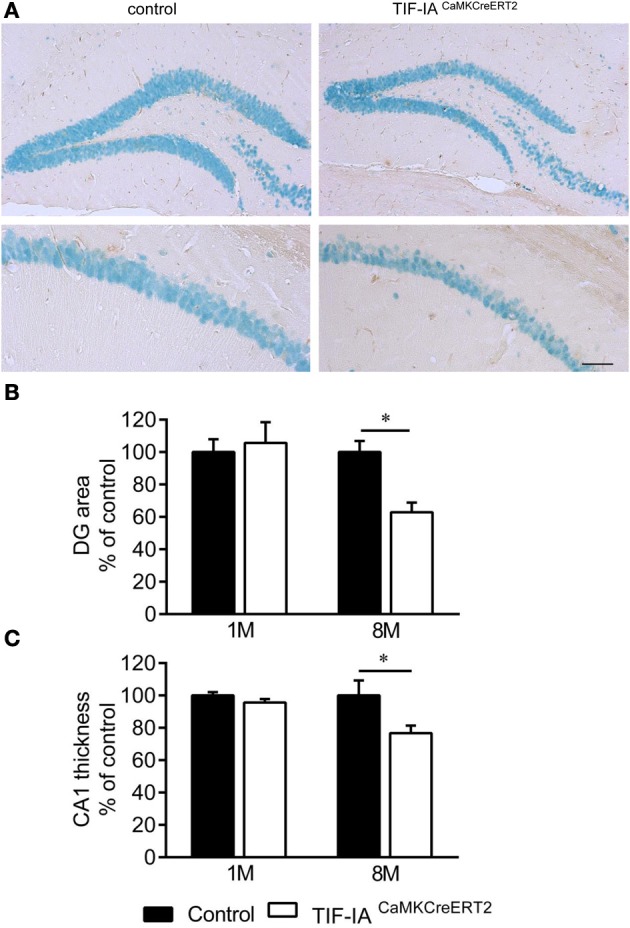
**Hippocampal neurons survive in DG and CA1 of TIF-IA^CaMKCreERT2^ mutants. (A)** Representative images of IHC with NeuN specific antibody in DG and CA1 of control and TIF-IA^CaMKCreERT2^ mutant mice 8 months after tamoxifen. **(B)** Quantification of DG area per section (% of control) in control and TIF-IA^CaMKCreERT2^ mutant mice 1 and 8 months after tamoxifen. **(C)** Quantification of CA1 thickness (% of control) in control and TIF-IA^CaMKCreERT2^ mutant mice 1 and 8 months after tamoxifen; *n* = 5. Error bars represent SEM. Scale bar: 120 μm. ^*^p < 0.05.

Inducible conditional ablation of the transcription factor TIF-IA by the Cre-loxP system impairs rRNA transcription and nucleolar integrity in the adult hippocampus (Parlato et al., 2008). TIF-IA mRNA and pre-rRNA level analyzed by qPCR were significantly reduced in the TIF-IA^CaMKCreERT2^ mutant mice 1 month after induction of the mutation by tamoxifen injection. GAPDH mRNA used as a control does not change (Figure 1A). rRNA transcription monitored by *in situ* hybridization detecting the nascent 47S pre-rRNA in hippocampal sections was strongly inhibited in the CA1 and DG 1 month after tamoxifen was given confirming our previous data (Parlato et al., 2008) (Figure 1B). Loss of TIF-IA nucleolar staining is evident in the mutants at the same stage; nonetheless without significantly altering mature 18S rRNA level (*p* = 0.58) (Figures 1B,C). Increased level of the tumor suppressor p53 is associated with death of hippocampal neurons 3 months after tamoxifen treatment (Parlato et al., 2008) suggesting its role in neuronal survival. Although the DG and the CA1 are significantly smaller than controls in TIF-IA^CaMKCreERT2^ mutant mice 8 months after tamoxifen treatment, hippocampal neurons do not undergo massive degeneration as for example dopaminergic neurons lacking TIF-IA (Figures 2A,B) (Rieker et al., 2011). In agreement with this, p53 immunoreactivity does not increase at later stages (data not shown). This initial analysis suggested that hippocampal neurons were more resistant to nucleolar stress.

**Figure 3:**
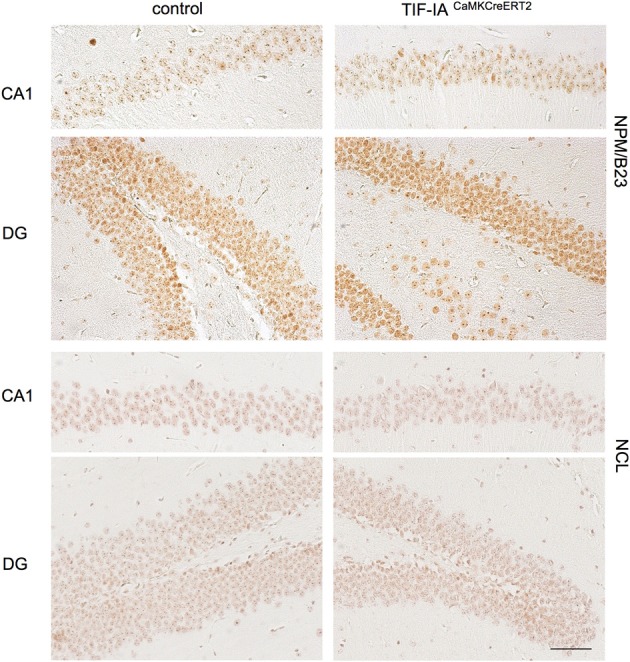
**Nucleolar integrity is altered in CA1 and DG of TIF-IA^CaMKCreERT2^ mutants**. Representative images of IHC with the nucleolar markers nuclephosmin (NPM/B23) and nucleolin (NCL) in control and TIF-IA^CaMKCreERT2^ mutant mice 1 month after tamoxifen showing reduced intensity of nucleolar staining of both proteins in the mutants. Scale bar: 60 μm.

To further explore these observations we investigated the distribution of the nucleolar protein nucleophosmin NPM/B23, commonly used as a marker of nucleolar stress upon various treatment and in different cellular contexts (Rubbi and Milner, 2003; Avitabile et al., 2011). Immunohistochemistry with NPM/B23 antibody shows that the controls are mainly characterized by an intense NPM/B23 staining in the nucleoli. In the DG increased NPM/B23 signal in the nucleoplasm is detectable in the subgranular zone (SGZ), occupied by sparse mitotic cells and it could be explained by loss of nucleolar integrity in dividing cells. One month after tamoxifen administration TIF-IA^CaMKCreERT2^ mutants showed reduced staining intensity in the nucleolus and increased signal in the nucleoplasm in comparison to controls (Figure 3). Notably, the nucleoplasmic distribution of NPM/B23 was more pronounced in the DG of the TIF-IA^CaMKCreERT2^ mutants (Figure 3).

To test whether other nucleolar proteins are affected at this stage, we analyzed the protein nucleolin (NCL), located in the granular component of the nucleolus and involved in various steps of ribosomal biogenesis (Abdelmohsen and Gorospe, 2012). NCL staining has been shown to decrease upon inhibition of rRNA biogenesis (Moore et al., 2013). Accordingly NCL intensity decreased in TIF-IA^CaMKCreERT2^ mutants supporting an initial induction of nucleolar stress 1 month after tamoxifen (Figure 3).

**Figure 4:**
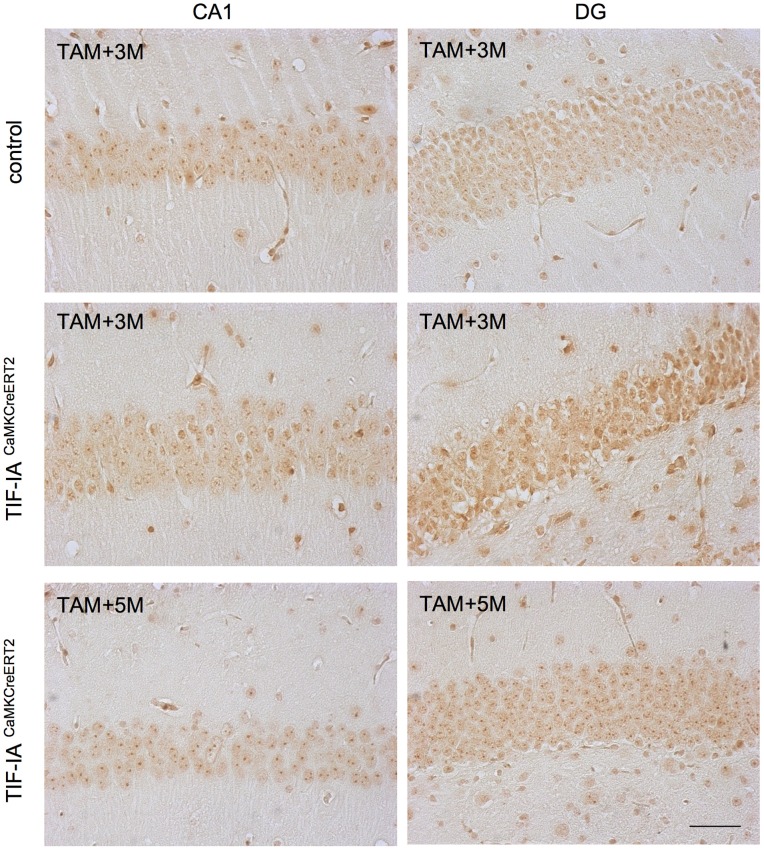
**Nucleolar integrity is restored in CA1 and DG of TIF-IA^CaMKCreERT2^ mutants**. Representative images of IHC with the nucleolar markers nuclephosmin (NPM/B23) in control and TIF-IA^CaMKCreERT2^ mutant mice 3 months after tamoxifen showing reduced intensity of nucleolar staining and re-localization to the nucleoplasm in the CA1 and mostly DG of mutants. Five months after tamoxifen TIF-IA^CaMKCreERT2^ show intense staining of NPM/B23 in the nucleoli. Scale bar: 60 μm.

We have previously shown that NPM/B23 re-localizes in the nucleoplasm of TIF-IA^CaMKCreERT2^ mutants 3 months after tamoxifen concomitant with increased neuronal death (Parlato et al., 2008). To monitor the progression of this phenomenon, we compared controls and TIF-IA^CaMKCreERT2^ mutants 2.5 and 5 months after tamoxifen (Figure 4). As previously shown, a consistent loss of nucleolar NPM was visible in CA1 and DG of TIF-IA^CaMKCreERT2^ mutants 2.5 months after tamoxifen. However, NPM nucleolar staining in both regions was similar in controls and TIF-IA^CaMKCreERT2^ mutants 5 months after tamoxifen (Figure 4), suggesting that nucleolar stress could be rescued at later stages in TIF-IA^CaMKCreERT2^ mutants.

**Figure 5:**
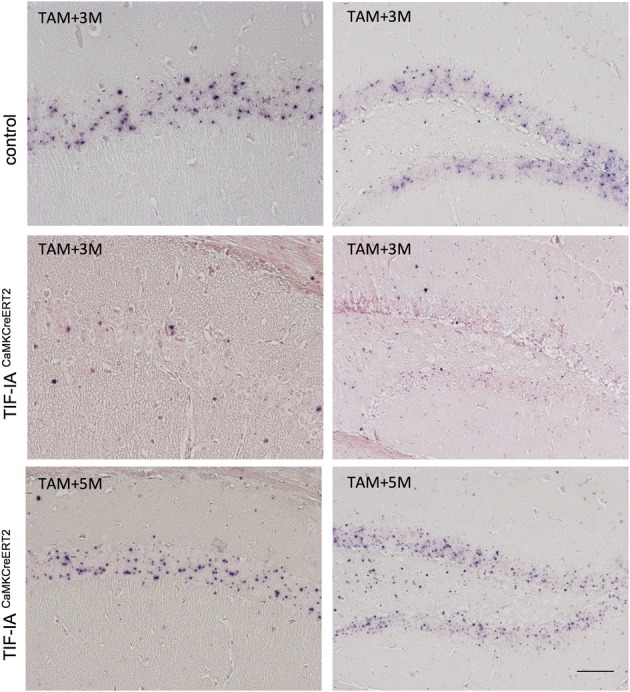
**pre-rRNA is restored in CA1 and DG of TIF-IA^CaMKCreERT2^ mutants**. Representative images of *in situ* hybridization performed in control and TIF-IA^CaMKCreERT2^ mutant mice 3 and 5 months after tamoxifen. Three months after tamoxifen TIF-IA^CaMKCreERT2^ mutant mice show loss of pre-rRNA in CA1 and DG in comparison to controls. Five months after tamoxifen TIF-IA^CaMKCreERT2^ show intense pre-rRNA staining in the nucleoli. The sections were slightly counterstained with nuclear fast red. Scale bar: 60 μm.

To further support this hypothesis we monitored rRNA transcription by *in situ* hybridization detecting 47S pre-rRNA in hippocampal sections 2.5 and 5 months after tamoxifen (Figure 5). This approach confirmed a loss of pre-rRNA at 2.5 months in the mutants, while at 5 months we found pre-rRNA positive cells in both CA1 and DG (Figure 5). These results indicated that pre-rRNA and nucleolar stress could be rescued over time in this model, possibly halting neurodegeneration.

**Figure 6:**
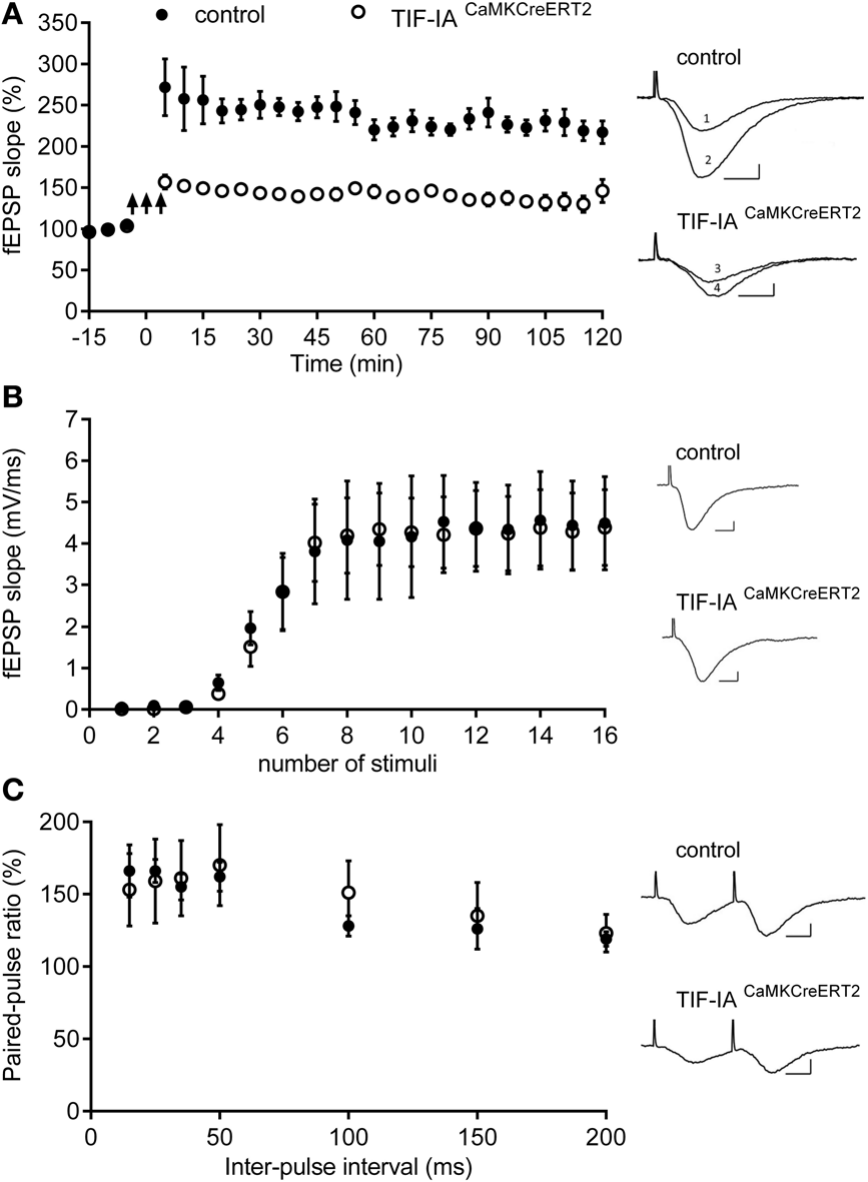
**TIF-IA^CaMKCreERT2^ mutant mice display hippocampal deficits in LTP. (A)** Tetanically induced LTP is lower in TIF-IA^CaMKCreERT2^ mutant mice (open circles) in comparison with control group (filled circles) 1 month after tamoxifen. Arrows mark the stimulation (3 trains of 100 Hz, 1 s stimulation separated by 3 min; *n* = 5, control; *n* = 6, mutant). Each point represents the average of 5 min recording from each animal group ± SEM. Right panels: superimposed representative fEPSP traces recorded from control and TIF-IA^CaMKCreERT2^ mutant mice 10 min before LTP induction (1 and 3, respectively) and 120 min after (2 and 4, respectively). Scale bars: 5 ms/2 mV. **(B)** Input-output (IO) curves representing the relationship between stimulus intensity and the slope of the fEPSP, did not differ significantly between control animals (filled circles; *n* = 5) and TIF-IA^CaMKCreERT2^ mutant mice (open circles; *n* = 6). Right panels: representative fEPSP traces with maximal slope, recorded from control (upper panel) and TIF-IA^CaMKCreERT2^ mutant mice (bottom panel). Scale bars: 5 ms/2 mV. **(C)** There were no differences in paired-pulse facilitation (PPF) ratios induced by delivering two stimuli with several different inter-stimulus intervals (15, 25, 35, 100, 150, and 200 ms) between control animals (filled circles; *n* = 5) and TIF-IA^CaMKCreERT2^ mutant mice (open circles; *n* = 6). The PPF ratio represents the slope of the second fEPSP divided by the slope of the first fEPSP presented in %. (Right panels) Representative fEPSP traces evoked by two stimuli with the same intensity and inter-stimulus interval 15 ms, recorded from control (upper panel) and TIF-IA^CaMKCreERT2^ mutant mice (bottom panel). Scale bars: 5 ms/2 mV.

To determine whether inhibition of rRNA synthesis observed 1 month after tamoxifen affects synaptic plasticity, we applied a tetanic stimulation protocol to Schaffer collaterals in controls and TIF-IA^CaMKCreERT2^ mutant mice at this early stage. Tetanic stimulation potentiated fEPSPs in controls and TIF-IA^CaMKCreERT2^ mutant mice were stable throughout the experiment and lasted at least 120 min. However, LTP in the mutant group was consistently lower (141 ± 7%; *n* = 6) in comparison with the control group (237 ± 15%; *n* = 5) (*p* = 0.001) (Figure 6). We also investigated the basic properties of synaptic transmission in both groups. There were no significant differences in the basic synaptic transmission (I-O curves) of fEPSP (Figure 6) between control and mutant mice. In addition we analyzed paired-pulse facilitation, a form of short-term plasticity. We found no differences in paired-pulse ratio between controls and TIF-IA^CaMKCreERT2^ mutants in all used inter-stimulus intervals (15, 25, 35, 50, 100, 150, and 200 ms).

### Sensorimotor responses, activity and anxiety are unaffected in TIF-IA^CaMKCreERT2^ mutant mice

To investigate the time-dependent consequences of nucleolar stress on hippocampal function, we analyzed behavioral, cellular, and molecular changes at different stages after induction of TIF-IA ablation.

Neurological examination starting 1 month after tamoxifen with an interval of 2–3 months revealed no difference between genotypes in home cage activity nor in functioning of all senses: olfactory, vision, hearing, and whisker movement. Motor coordination and balance were measured by the Rotarod test. TIF-IA^CaMKCreERT2^ mutant mice retained normal motoric functions and coordination as they showed similar endurance to controls (Table 1). In new, open space (Open Field test) TIF-IA^CaMKCreERT2^ mutant mice were as active as controls. During 5-min test controls and mutants covered similar distance and number of zones (Table 1). Also habituation to a new environment measured as a distance traveled in each minute of test was not changed in the mutant mice (Table 1). Anxiety to open, elevated space was assessed by 5-min exposure to Elevated Plus Maze. Mutant and control mice spent both more time in the closed arms than in the open arms of the apparatus showing similar level of anxiety (Table 1). This observation was confirmed by assessing anxiety level in the open field as time spent in the central vs. the periphery zone. Independently of the mutation, the mice spent less than 10% of time in the aversive zone of open field showing strong preference for the periphery zone (Table 1).

**Table 1:** **Statistical analysis of the behavioral tests**.

**Test**	**Parameter**	**Difference between genotypes: control vs. mutant mice**		
		**5W after TAM control: *n* = 16 mutant: *n* = 15**	**3M after TAM control: *n* = 6 mutant: *n* = 5**	**6M after TAM control: *n* = 6 mutant: *n* = 7**
Rotarod	Time to fall (s)	One-way ANOVA	One-way ANOVA	One-way ANOVA
		*F*_(1, 27)_ = 1.855; *P* level = 0.184	*F*_(1, 7)_ = 1.888; *P* level = 0.677	*F*_(1, 9)_ = 0.106; *P* level = 0.920
**ACTIVITY AND HABITUATION IN OPEN FIELD**				
Open field	Total distance moved (cm)	One-way ANOVA	One-way ANOVA	One-way ANOVA
		*F*_(1, 27)_ = 2.223; *P* level = 0.147	*F*_(1, 7)_ = 0.264; *P* level = 0.623	*F*_(1, 9)_ = 2.18; *P* level = 0.174
	Number of zones crossed	One-way ANOVA	One-way ANOVA	One-way ANOVA
		*F*_(1, 27)_ = 3.181; *P* level = 0.086	*F*_(1, 7)_ = 0.628; *P* level = 0.454	*F*_(1, 9)_ = 1.55; *P* level = 0.244
	Velocity (cm/s)	One-way ANOVA	One-way ANOVA	One-way ANOVA
		*F*_(1, 27)_ = 2.247; *P* level = 0.145	*F*_(1, 7)_ = 0.068; *P* level = 0.801	*F*_(1, 9)_ = 2.18; *P* level = 0.174
	Distance moved in subsequent minutes	ANOVA with repeated measures	ANOVA with repeated measures	ANOVA with repeated measures
		*F*_(1, 27)_ = 1.563; *P* level = 0.221	*F*_(1, 7)_ = 0.240; *P* level = 0.636	*F*_(1, 9)_ = 2.823; *P* level = 0.121
**ANXIETY IN ELEVATED PLUS MAZE AND OPEN FIELD**				
Elevated plus maze	Time (%) spent in zones of apparatus	One-way ANOVA	One-way ANOVA	One-way ANOVA
		closed arms *F*_(1, 27)_ = 0.164; *P* level = 0.688	closed arms *F*_(1, 7)_ = 4.031; *P* level = 0.085;	closed arms *F*_(1, 9)_ = 0.071; *P* level = 0.795;
		open arms *F*_(1, 27)_ = 0.069; *P* level = 0.794	open arms *F*_(1, 7)_ = 2.941; *P* level = 0.130	open arms *F*_(1, 9)_ = 0.214; *P* level = 0.887
Open field	Time (%) spent in center vs. periphery	One-way ANOVA	One-way ANOVA	One-way ANOVA
		*F*_(1, 9)_ = 0.472; *P* level = 0.509	*F*_(1, 27)_ = 0.235; *P* level = 0.631	*F*_(1, 12)_ = 0.0004; *P* level = 0.983
**LEARNING AND MEMORY IN MORRIS WATER MAZE**				
Visible platform test	Latency (s)	One-way ANOVA	One-way ANOVA	One-way ANOVA
		*F*_(1, 27)_ = 2.223; *P* level = 0.147	*F*_(1, 7)_ = 0.304; *P* level = 0.98	*F*_(1, 9)_ = 2.18; *P* level = 0.174
	Swim speed (cm/s)	One-way ANOVA	One-way ANOVA	One-way ANOVA
		*F*_(1, 27)_ = 1.066; *P* level = 0.310	*F*_(1, 7)_ = 0.110; *P* level = 0.747	*F*_(1, 9)_ = 0.015; *P* level = 0.904
Training to platform location 1, days 1–4	Latency (s)	One-way ANOVA with repeated measures	One-way ANOVA with repeated measures	One-way ANOVA with repeated measures
		*F*_(1, 27)_ = 4.602; *P* level = 0.041	*F*_(1, 7)_ = 1.805; *P* level = 0.221	*F*_(1, 9)_ = 0.001; *P* level = 0.975
	Swim speed (cm/s)	One-way ANOVA with repeated measures	One-way ANOVA with repeated measures	One-way ANOVA with repeated measures
		*F*_(1, 27)_ = 1.186; *P* level = 0.285	*F*_(1, 7)_ = 0.585; *P* level = 0.464	*F*_(1, 9)_ = 0.040; *P* level = 0.846
Training to platform location 2	Latency (s)	One-way ANOVA	One-way ANOVA	One-way ANOVA
		*F*_(1, 27)_ = 0.724; *P* level = 0.012	*F*_(1, 7)_ = 10.808; *P* level = 0.009	*F*_(1, 9)_ = 0.671; *P* level = 0.434
	Swim speed (cm/s)	One-way ANOVA	One-way ANOVA	One-way ANOVA
		*F*_(1, 27)_ = 1.180; *P* level = 0.286	*F*_(1, 7)_ = 0.099; *P* level = 0.761	*F*_(1, 9)_ = 0.061; *P* level = 0.809
Test 1	Time in target zone (%)	One-way ANOVA	One-way ANOVA	One-way ANOVA
		*F*_(1, 27)_ = 0.666; *P* level = 0.422	*F*_(1, 7)_ = 0.259; *P* level = 0.626	*F*_(1, 9)_ = 0.812; *P* level = 0.391
Test 2	Test 2, time in target zone (%)	One-way ANOVA	One-way ANOVA	One-way ANOVA
		*F*_(1, 27)_ = 0.019; *P* level = 0.892	*F*_(1, 7)_ = 0.011; *P* level = 0.920	*F*_(1, 9)_ = 0.463; *P* level = 0.513

### Loss of TIF-IA enhances spatial learning and re-learning in morris water maze

**Figure 7:**
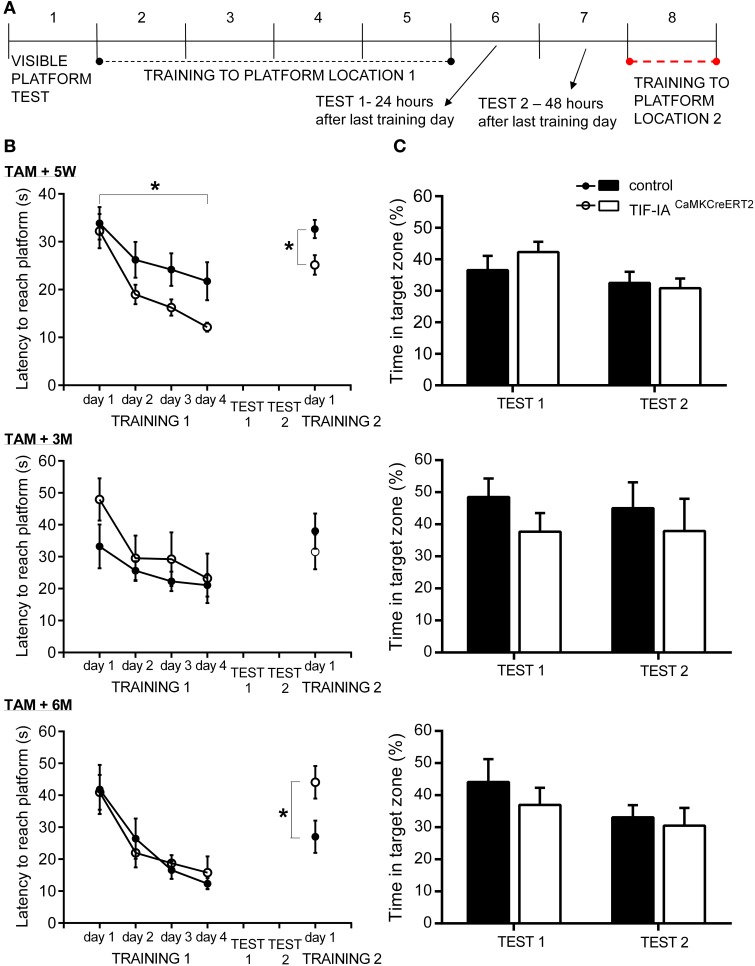
**TIF-IA^CaMKCreERT2^ mutant mice show enhanced learning. (A)** Morris water maze procedure. Trials order in subsequent days. **(B)** Spatial learning in Morris water maze 5 weeks, 3 and 6 months after tamoxifen. Time to find a hidden platform in 4-day learning and 1-day re-learning is shown. Enhanced learning abilities in mutant compared to control mice (*p* < 0.05) were observed 5 weeks after induction of mutation. Deterioration in 1-day re-learning task was found in mutants 6 months after tamoxifen. No differences in swim speed were found. **(C)** Spatial memory tests in Morris water maze 5 weeks, 3 and 6 months after tamoxifen. Relative time of swimming in target quadrant (%) is shown. Memory tests performed 24 h (Test 1) as well as 48 h (Test 2) after the last training day, showed memory for platform location in control and mutant mice tested in each time points after induction of mutation. Randomness level (25%) is indicated by dotted line. ^*^p < 0.05.

In the visible platform test, all mice showed no motivational, visual, or motoric problems in reaching the platform. Both TIF-IA^CaMKCreERT2^ mutant and control mice found the visible platform in less than 15 s (Table 1). Spatial learning was assessed during the training phase by latency to reach the platform (Figure 7A) and swim speed (Table 1). While swim speed was similar between genotypes, we found differences in time to find the hidden platform. Five weeks after induction of TIF-IA mutation, we observed a better performance of TIF-IA^CaMKCreERT2^ in comparison to controls and to other time points (Figure 7B, Table 1). In the first day of training both mutant and control mice required similar time to find the platform. However, from the 2nd to 4th day of training, the TIF-IA^CaMKCreERT2^ mutant mice were able to reach the platform in less than 20 s, while the controls needed longer time. Also in re-learning task, the mutants found the platform in a new location (position 2) faster than control mice (Figure 7B). However, mutants tested 3 and 6 months after induction of the mutation, improved their time to find a platform similarly to controls. No differences were found in 1-day of reversal learning between TIF-IA^CaMKCreERT2^ mutant mice and controls 9 weeks after induction of the mutation. However, 6 months after tamoxifen, TIF-IA^CaMKCreERT2^ mutant mice showed worsened re-learning abilities in comparison to control mice (training 2, Figure 7A). In this case the mice experienced 1 day of re-training to learn a new platform position (position 2). TIF-IA^CaMKCreERT2^ mutant mice spent significantly longer time to find the platform new location than control mice (Figure 7B, Table 1).

Spatial memory was evaluated in 2 probe trials (Figure 7A). The platform was removed and the time spent in different quadrants of the pool was measured. In the first probe trial (24 h after the last training day) both mutant and control mice showed memory of the platform position, as they selectively searched for a platform in the proper quadrant for longer than 25% (chance level) of total time swimming. 48 h after the last training day, in the second probe trial, all mice spent more than 25% of time in the proper quadrant (Figure 7C). No differences between genotypes were found in quadrant preferences at any time points after ablation of TIF-IA gene (Table 1).

Sexual dimorphism in spatial learning has been observed in several studies, in particular mouse males showed reduced latencies than females at the initial learning stages (Schmidt et al., 2009; Chow et al., 2013). To address this aspect in control and TIF-IA^CaMKCreERT2^ mutants we analyzed gender differences after tamoxifen treatment (Table 2). Despite some apparent trends in “Training to platform location 1,” based on Two-Way ANOVA we could not detect any significant differences between males and females; for example over 1–4 training days we observed the following: Latency (s): “genotype” *F*_(1, 27)_ = 4.602; *p* = 0.041, “sex” *F*_(1, 27)_ = 2.426; *p* = 0.130, “Interaction sex^*^genotype” *F*_(1, 27)_ = 2.115; *p* = 0.157.

**Table 2:** **Analysis of gender differences in control and TIF-IA^CaMKCreERT2^ 5 weeks after tamoxifen treatment**.

**Test**	**Parameter**	**5W after TAM Controls, n = 16: 8 females, 8 males Mutants: n = 15: 5 females, 10 males**	
Visible platform test	**Latency (s)**	**Average**	**SEM**
	Control females	14.09	3.53
	Control males	13.5	5
	Mutant females	12.81	6.49
	Mutant males	12.37	9.64
	**Swim speed (cm/s)**	**Average**	**SEM**
	Control females	19.9	1.09
	Control males	20.45	0.56
	Mutant females	20.88	0.95
	Mutant males	18.54	0.59
Training to platform location 1, day 4	**Latency (s)**	**Average**	**SEM**
	Control females	27.79	7.33
	Control males	15.74	2.06
	Mutant females	13.03	1.48
	Mutant males	11.72	1.21
	**Swim speed (cm/s)**	**Average**	**SEM**
	Control females	20.71	1.09
	Control males	21.25	0.56
	Mutant females	21.68	0.95
	Mutant males	19.34	0.59
Training to platform location 2	**Latency (s)**	**Average**	**SEM**
	Control females	29.3	1.08
	Control males	33.99	3.076
	Mutant females	22.57	3.60
	Mutant males	27.71	2.49
	**Swim speed (cm/s)**	**Average**	**SEM**
	Control females	20.30	0.88
	Control males	21.64	0.83
	Mutant females	21.66	1.15
	Mutant males	19.18	0.67
Test 1	**Time in target zone (%)**	**Average**	**SEM**
	Control females	28.73	6.15
	Control males	44.38	5.76
	Mutant females	36.83	7.55
	Mutant males	45.03	3.19
Test 2	**Time in target zone (%)**	**Average**	**SEM**
	Control females	32.81	6.13
	Control males	32.15	4.07
	Mutant females	30.99	6.40
	Mutant males	30.73	3.66

### Hippocampal neurons adopt early compensatory mechanisms in response to nucleolar stress

**Figure 8:**
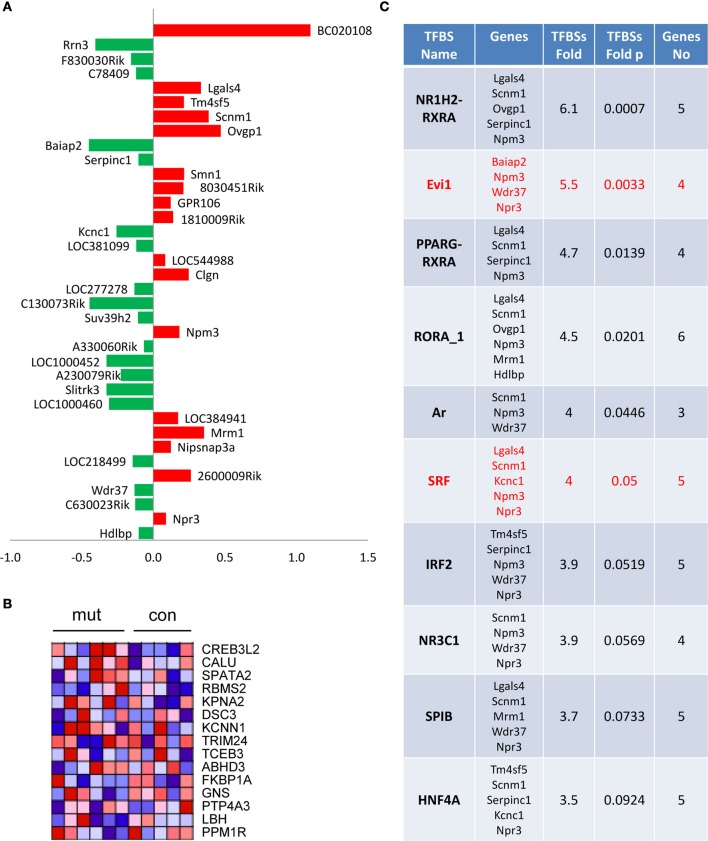
**Moderate effects on gene expression in TIF-IA^CaMKCreERT2^ mutant mice**. One month after tamoxifen treatment. **(A)** Identification of 36 transcripts at unadjusted *p* < 0.001 and their fold change vs. control group; **(B)** Heat map visualization of gene set enrichment analysis (GSEA) reveals changes in genes associated with vincristine resistance; **(C)** Identification of enriched putative transcription factor binding sites performed using cREMaG database.

To further investigate the early molecular alterations caused by inhibition of rRNA transcription, we analyzed changes in gene expression 1 month after tamoxifen injection in control and TIF-IA^CaMKCreERT2^ mutants (Figure 8). Gene expression profiling analysis showed moderate effects of the mutation on gene expression (36 transcripts differentially expressed at *p* < 0.001, as compared to the control group). Interestingly, among these transcripts we found a significant decrease in the TIF-IA/Rrn3 mRNA. In line with normal neuronal survival at this stage we did not detect any genes related to neuronal death, inflammation or gliosis (Figure 8A). Nevertheless, GSEA revealed similarities between mutation-induced changes in gene expression and some of the previously reported expression patterns elicited by cellular stress (Figure 8B). Additionally, enrichment in putative serum response factor (SRF) binding sites in the promoters of the genes with changed expression was observed, indicating potential adaptation in the mitogen-activated protein kinase cascade which could be regarded as a pro-survival response (Figure 8C).

### Hippocampal neurons respond to nucleolar stress by activation of the mTOR signaling

**Figure 9:**
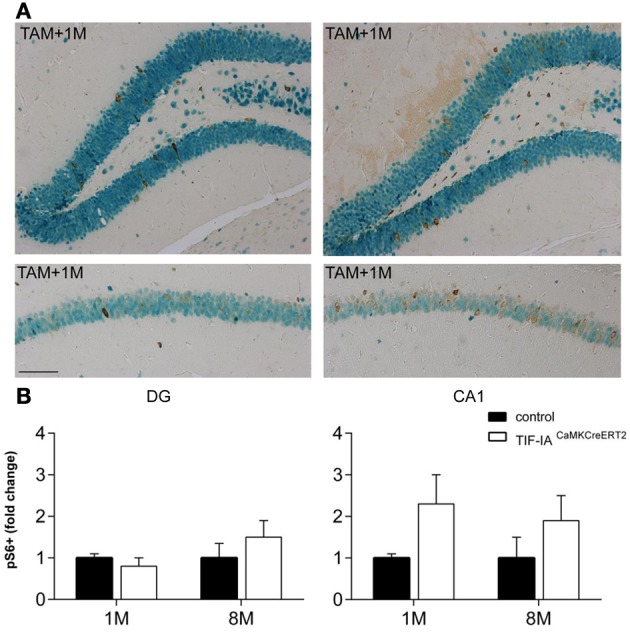
**mTOR signaling is active in control and TIF-IA^CaMKCreERT2^ mutant mice. (A)** Representative images of the immunostaining with phospho-S6 (brown) and NeuN (green) antibodies in DG and CA1 on paraffin sections of control and TIF-IA^CaMKCreERT2^ mutant mice 1 month after tamoxifen. **(B)** Quantification of phospho-S6 positive neurons 1 and 8 months after tamoxifen in DG (left panel) and CA1 (right pane); *n* = 5. Scale bar: 120 μm.

Because we have recently reported that loss of TIF-IA leads to downregulation of mTOR activity and neurodegeneration of dopaminergic neurons (Rieker et al., 2011), we have analyzed mTOR activity in CA1 and DG of TIF-IA^CaMKCreERT2^ mutants and controls 1 and 8 months after tamoxifen to establish how this pathway responds to nucleolar stress in hippocampal neurons. Phosphorylation of the ribosomal S6 protein is a well-known marker of mTOR activation. Therefore, we have performed immunohistochemistry with NeuN and pS6 specific antibodies and counted the number of NeuN positive cells also labeled by phosphoS6 (Figure 9). In contrast to dopaminergic neurons lacking TIF-IA, mTOR activity was not down-regulated at any stages in hippocampal neurons, and it rather appeared up-regulated in TIF-IA^CaMKCreERT2^ mutants (Figure 9B). This analysis suggests that increased levels of pS6 and mTOR activity may be a compensatory response to nucleolar stress in hippocampal neurons.

### Neurogenesis increases in TIF-IA^CaMKCreERT2^ mutants

**Figure 10:**
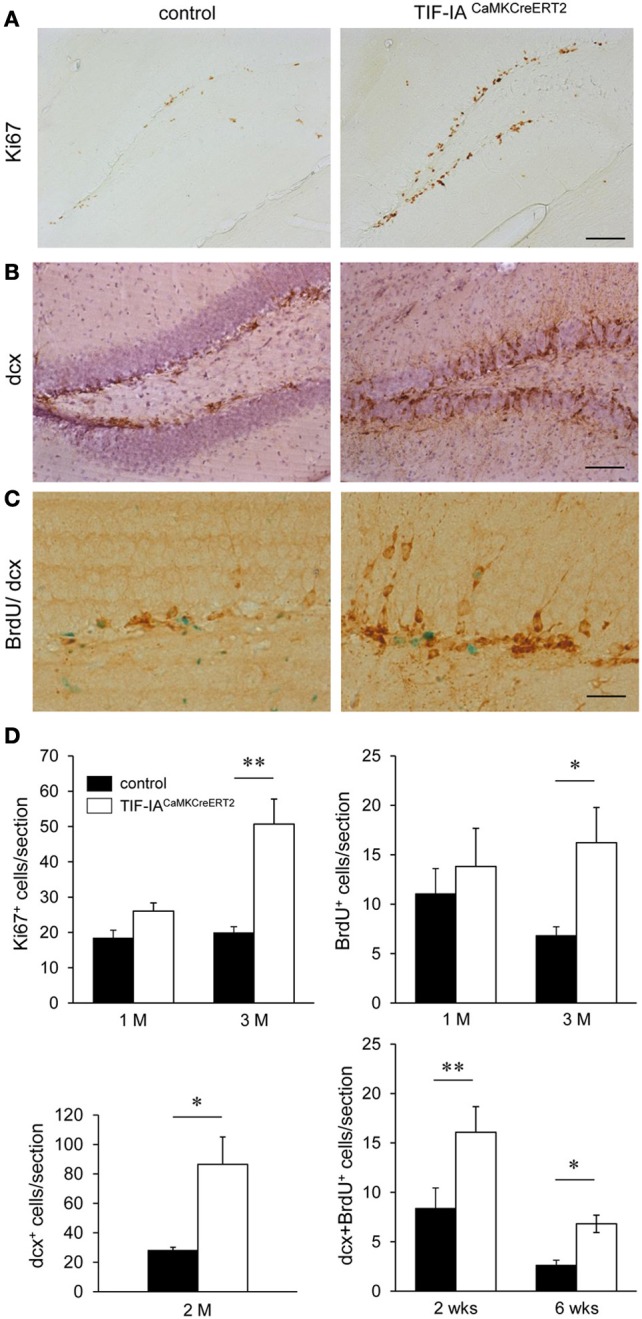
**Increased neurogenesis in TIF-IA^CaMKCreERT2^ mutant mice. (A)** Analysis of Ki67 positive cells in the DG 3 months after tamoxifen in controls and TIF-IA^CaMKCreERT2^ mutants. **(B)** Representative images of doublecortin positive cells in the DG 3 months after tamoxifen. Hematoxylin counterstaining has been used to visualize the nuclei. **(C)** Control and mutant mice were injected for 5 days with BrdU 2.5 months after tamoxifen. Doublecortin (dcx) positive (brown) BrdU retaining (green) cells were counted 2 and 6 weeks after BrdU injections. **(D)** Quantification of Ki67 positive, and BrdU positive cells (2 h after single BrdU injection) 1 and 3 months after tamoxifen (upper panels). Quantification of the increased dcx positive cells shows significant increase 2 months after tamoxifen. Quantification of doublecortin positive cells retaining BrdU staining 2 and 6 weeks after BrdU injections shows significant increase in the TIF-IA^CaMKCreERT2^ mutant mice (lower panels); *n* = 5–6. Scale bars: **(A,B)** 120 μm; **(C)** 60 μm. ^*^p < 0.05; ^**^p < 0.01.

Adult neurogenesis in the SGZ of the hippocampus occurs in response to environmental changes and during disease progression (Lledo et al., 2006). As nucleolar stress in the TIF-IA^CaMKCreERT2^ mutants does not lead to complete loss of hippocampal neurons or of their function (Figures 2, 7), we analyzed neurogenesis in the hippocampus of controls and TIF-IA^CaMKCreERT2^ mutants at different stages after tamoxifen. By immunohistochemistry based on the proliferation marker Ki67 we detected increased proliferating cells in the DG of TIF-IA^CaMKCreERT2^ mutants 2.5–3 months after tamoxifen (Figures 10A,D). In this set of mice DG area was not significantly different in the mutants 1 month after tamoxifen (89% of controls, *p* = 0.3) and it was reduced 3 months after tamoxifen (80% of controls, *p* = 0.0006). We showed increased proliferating cells also by normalization of the Ki67 labeled cells to the DG area of the respective section (mm^2^) (control 1 M: 88.5 ± 5.3; mutant 1 M: 144 ± 21, *p* = 0.06 and control 3 M: 98.7 ± 15; mutant 3 M: 311.9 ± 44, *p* = 0.01). This increase was confirmed by the higher number of bromodeoxyuridine (BrdU) positive cells in TIF-IA^CaMKCreERT2^ mutants at the same stage (Figure 10D). Increased neurogenesis was supported by the significantly higher number of doublecortin positive cells, a marker of immature neurons in TIF-IA^CaMKCreERT2^ mutants (Figures 10B,D).

In adult mice the maturation of newly generated hippocampal neurons in the DG is complete by 4 months of age (van Praag et al., 2002; Laplagne et al., 2006); nevertheless neuronal properties emerge between 4 and 8 weeks. To establish whether increased BrdU labeling reflects increased neurogenesis we analyzed the number of doublecortin positive cells retaining BrdU staining 2 and 6 weeks after labeling. The increased number of double positive cells in the TIF-IA^CaMKCreERT2^ mutants (Figures 10C,D) indicated higher generation of immature neurons. We showed similar results by normalization of the labeled cells to the DG area of the respective section (mm^2^) (control 2 weeks: 43.5 ± 7.5; mutant 2 weeks: 104 ± 8, *p* = 0.001 and control 6 weeks: 13.7 ± 2.3; mutant 6 weeks: 40.3 ± 5.7, *p* = 0.01).

## Discussion

Inhibition of rRNA synthesis and nucleolar morphology is associated with aging and several neurological and psychiatric disorders linked to altered protein synthesis including neurodegenerative diseases, autism spectrum disorders (ASD), and depression (Hetman and Pietrzak, 2012; Parlato and Kreiner, 2013). In this study we showed that hippocampal neurons adopt a variety of cellular and molecular strategies to preserve their function in response to inhibition of rRNA transcription and nucleolar stress. These adaptive mechanisms include increased neurogenesis in the DG, early activation of neuroprotective pathways and late recovery of rRNA transcription.

The TIF-IA^CaMKCreERT2^ mutants represent a unique model to investigate the effects of perturbed nucleolar activity on protein translation and synaptic transmission at different stages. We identified three major stages: an initial phase characterized by pre-rRNA transcription inhibition and incipient nucleolar stress, reduced LTP but no effects on neuronal survival and improved learning performance (1 month after tamoxifen), a second phase characterized by nucleolar stress linked to a peak of neurodegeneration and neurogenesis (3 months after tamoxifen), and a third phase in which nucleolar function and integrity is restored and neurodegeneration is halted (5–8 months after tamoxifen).

In particular, concomitant with the first phase we observed shorter escape latencies in TIF-IA^CaMKCreERT2^ mutants than controls during acquisition and reversal of Morris water maze. At the same stage the mutants also showed enhanced (re-)learning abilities in spatial tasks. However, in the probe tests TIF-IA^CaMKCreERT2^ mice performed as well as controls. Interestingly, improved learning performance was not correlated with enhanced synaptic plasticity, as LTP was decreased compared to the control group indicating that TIF-IA may play a role in the establishment of LTP. Although LTP is a cellular mechanism of the learning process and its enhancement in general accompanies learning, some exceptions have been reported in line with our results. A similar phenomenon to that observed in TIF-IA^CaMKCreERT2^ mice was for example documented in the GluR1 knock-out mice. In this model LTP was absent at the synapses formed between CA3-CA1, whereas spatial learning tested in the Morris water maze was not affected (Zamanillo et al., 1999; Reisel et al., 2002; Sanderson et al., 2008). In TIF-IA^CaMKCreERT2^ model the molecular basis of this dissociation between LTP and improved learning requires further investigation.

Enhanced adult hippocampal neurogenesis could account for the learning and memory similarities between controls and mutant mice observed in the second phase. It has been previously shown that neurogenesis and learning of water maze task are correlated (Kempermann and Gage, 2002). New neurons in adult hippocampus may provide better flexibility for the acquisition of spatial platform position and for learning a changed platform position (reversal) (Garthe et al., 2009). This flexibility, however, was not sustained over time, as TIF-IA^CaMKCreERT2^ mice 6 months after tamoxifen show deterioration of reversal learning.

In addition the TIF-IA^CaMKCreERT2^ mutants allow to explore how newly generated neurons adapt to the local environment. In other mouse models of hippocampal neurodegeneration such as the conditional mutant mice lacking the pro-survival transcription factors CREB (cAMP-responsive element binding protein) and CREM (cAMP-responsive element modulator) in the postnatal forebrain (Mantamadiotis et al., 2002), hippocampal neurons undergo massive neurodegeneration, despite increased neurogenesis in the DG (Luzzati et al., 2011). In fact, the newly generated neurons are also affected by the constitutive loss of CREB upon expression of the Cre recombinase leading to their death. The TIF-IA^CaMKCreERT2^ mutants are inducible upon injection of tamoxifen; therefore the newly generated neurons do not carry the TIF-IA mutation and are provided with an intact RNA polymerase I transcription apparatus. Certainly it is puzzling how neurons can initially survive and function without pre-rRNA synthesis and how pre-rRNA synthesis is restored. Although further studies are required to establish whether increased neurogenesis accounts for keeping intact spatial learning and memory in TIF-IA^CaMKCreERT2^ mutants and nucleolar function, other scenarios should be envisaged.

An intriguing hypothesis is that ribosomes are transported at the degenerating synapses by either other neurons or glial cells. This mechanism has been recently described between Schwann cells and regenerating axons, suggesting a potential compensatory mechanism in response to rRNA synthesis perturbation (Court et al., 2008, 2011).

Another explanation of the confined nucleolar effects on neuronal survival and function in the hippocampus could be that the DG is a region with low activity in which only a low proportion of neurons are active. On the contrary immature neurons in adult hippocampus show increased activity and high excitability as during development (Piatti et al., 2013). Thus, the combination of these two factors could account for hippocampal adaptation.

Concomitantly, loss of TIF-IA could be compensated by the induction of a transcription factor playing a similar role in the regulation of RNA Pol I activity and/or mechanisms altering the turn-over of ribosomes and pre-rRNA processing could participate to the maintenance of protein synthesis (Granneman and Baserga, 2005; Kopp et al., 2007). A possible candidate is the NPM/B23 itself; in response to inhibition of RNA polymerase I activity NPM/B23 has been reported to complex with the mRNA-binding proteins hnRNPU and hnRNPA1 in the cytoplasm influencing rRNA processing and sustaining cell survival (Yao et al., 2010). Moreover NPM/B23 may regulate rRNA transcription by its association with the rDNA promoter and function as a histone chaperone (Murano et al., 2008). Decreased level of NPM protein independently of mRNA differences has been reported as a consequence of kainic-acid induced excitoxicity in hippocampal CA1 and NPM/B23 overexpression is neuroprotective against this insult, suggesting an important function in survival of hippocampal neurons (Marquez-Lona et al., 2012). We cannot exclude that reduced NPM/B23 is accompanied by reduced protein expression in the nucleoli in TIF-IA^CaMKCreERT2^ mutants.

Interestingly, nucleolar hypertrophy is linked to neuronal resistance to neurotoxic β-amyloid deposits and Aβ plaques in the CA1 of asymptomatic AD subjects; on the contrary in AD there is a significant atrophy of the nucleoli in the CA1 (Iacono et al., 2008). These examples indicate multiple mechanisms sustaining nucleolar activity in hippocampal neurons under stress that require further investigation.

At the intracellular level, the upregulation of the MAPK and mTOR pathways might limit the effects of disrupted rRNA transcription steering the response toward maintenance of functional protein synthesis and neuronal survival and activity (Mendoza et al., 2011). Because here we monitor S6 phosphorylation at Ser235/236 that are target sites common to RSK and S6K, we cannot exclude that upregulation of Ras-ERK pathway promotes the compensatory response, as also suggested by the gene expression profiling analysis. Upstream regulators of mTOR include growth factors, nutrients, energy and stress and a wired network involving PI3K/Akt and Ras/ERK to control translation, ribosome biogenesis, autophagy, transcription, and metabolism (Wullschleger et al., 2006). The molecular basis of enhanced mTOR and/or ERK activity in TIF-IA^CaMKCreERT2^ mice remains as of yet unknown. Nevertheless, enhanced mTOR activity is common to ASD (Kelleher and Bear, 2008). Interestingly, genetically increasing the levels of eukaryotic translation initiation factor 4E (eIF4E) in mice results in exaggerated cap-dependent translation and behaviors reminiscent of autism (Santini et al., 2013). Future research should address how nucleolar activity is regulated in the pathophysiology of ASD and other brain disorders in which global or local protein synthesis is up-regulated.

Certainly, the mechanisms enabling neurons to survive despite inhibition of pre-rRNA synthesis will help to better understand neuronal stress response and defense strategies. Notably, although TIF-IA deficient dopaminergic and dopaminoceptive neurons undergo virtually complete loss, these neurons still survive several months without pre-rRNA synthesis, despite a strong nucleolar stress response triggering high level of p53 (Rieker et al., 2011; Kreiner et al., 2013). Nucleolar stress in dopaminoceptive neurons leads for example to autophagy mediated by p53-mediated mTOR inhibition, extending neuronal survival (Kreiner et al., 2013).

In conclusion, our data reveal an intricate series of events potentially relevant for therapeutic applications in neurological and psychiatric disorders related to disturbances of the hippocampal translational machinery.

### Conflict of interest statement

The authors declare that the research was conducted in the absence of any commercial or financial relationships that could be construed as a potential conflict of interest.
